# Epidermal Growth Factor Receptor in Prostate Cancer Derived Exosomes

**DOI:** 10.1371/journal.pone.0154967

**Published:** 2016-05-06

**Authors:** Geetanjali Kharmate, Elham Hosseini-Beheshti, Josselin Caradec, Mei Yieng Chin, Emma S. Tomlinson Guns

**Affiliations:** 1 Department of Urologic Sciences, University of British Columbia, Vancouver, Canada; 2 Vancouver Prostate Centre, University of British Columbia, Vancouver, Canada; 3 Department of Experimental Medicine, University of British Columbia, Vancouver, Canada; Southern Illinois University School of Medicine, UNITED STATES

## Abstract

Exosomes proteins and microRNAs have gained much attention as diagnostic tools and biomarker potential in various malignancies including prostate cancer (PCa). However, the role of exosomes and membrane-associated receptors, particularly epidermal growth factor receptor (EGFR) as mediators of cell proliferation and invasion in PCa progression remains unexplored. EGFR is frequently overexpressed and has been associated with aggressive forms of PCa. While PCa cells and tissues express EGFR, it is unknown whether exosomes derived from PCa cells or PCa patient serum contains EGFR. The aim of this study was to detect and characterize EGFR in exosomes derived from PCa cells, LNCaP xenograft and PCa patient serum. Exosomes were isolated from conditioned media of different PCa cell lines; LNCaP xenograft serum as well as patient plasma/serum by differential centrifugation and ultracentrifugation on a sucrose density gradient. Exosomes were confirmed by electron microscopy, expression of exosomal markers and NanoSight^™^ analysis. EGFR expression was determined by western blot analysis and ELISA. This study demonstrates that exosomes may easily be derived from PCa cell lines, serum obtained from PCa xenograft bearing mice and clinical samples derived from PCa patients. Presence of exosomal EGFR in PCa patient exosomes may present a novel approach for measuring of the disease state. Our work will allow to build on this finding for future understanding of PCa exosomes and their potential role in PCa progression and as minimal invasive biomarkers for PCa.

## Introduction

Prostate cancer (PCa) is the second leading cause of death among Western males. Preserved activity of the androgen receptor is the main driver for PCa progression and metastasis [1, 2]. Early stage PCa is curable, however, one-third of the cases progress to a more aggressive PCa with poor patient survival [3]. Despite the availability of several therapeutic strategies, targeting metastases and managing disease relapse remains a challenge. Hence, successful early detection of PCa is of great importance. Apart from the commonly used diagnostic procedures/tests such as prostate specific antigen (PSA) testing and digital rectal examination [4], a critical need remains for us to discover new biomarkers and develop a more sensitive yet minimally invasive tests for better and early diagnosis of PCa.

There is growing evidence suggesting that cancer cells release microvesicles of 30–100 nm in diameter known as ‘*exosomes’*, and that exosomes are readily found in biological fluids including plasma, serum, malignant ascites, urine and breast milk [5, 6]. Exosomes are derived from late endosomes known as multivesicular bodies (MVBs) and are released upon fusion of the MVBs with the plasma membrane [7]. Exosomes contain unique protein and RNA cargo that are released into the cellular microenvironment and thus can promote cell-cell communication in addition to other mechanisms [8, 9]. Recently, our lab and others have demonstrated that benign as well as PCa cells with or without androgen receptor (AR) release exosomes [9, 10]. Additionally, a comprehensive lipid and proteomic analysis revealed distinct differences in protein profiles of exosomes derived from benign as compared to malignant PCa cell lines [9, 11, 12]. Studies have shown that exosomes contain membrane-associated proteins which act as mediators of cell growth and likely to confer cellular phenotypic change via cell-cell communication [13–15]. However, the role of component membrane-associated growth factor receptors of exosomes as mediators of cell proliferation and invasion remains unexplored.

In addition to androgens, prostate growth and function is in-part regulated by several growth factors and their cognate receptors, one of which is the epidermal growth factor and its receptor (EGFR)[16, 17]. EGFR is a 170 kDa proto-oncogene and transmembrane receptor which is typically over-expressed in various malignancies including PCa [18]. Ligand binding to EGFR induces dimerization, phosphorylation and internalization of the EGFR which then trigger a network of intracellular signalling pathways, resulting in DNA synthesis, cell proliferation, migration and adhesion [18]. It has been shown that nearly 30% of PCa cases overexpress EGFR and that deregulation of EGFR-mediated signaling pathways is associated with poor clinical outcomes [19, 20]. Although EGFR is identified as an important anti-tumor target, therapies against EGFR using small tyrosine kinase inhibitors such as Gefitinib, Lapatinib and Erlotinib have been shown to have limited effectiveness in PCa [21–23]. While the intracellular trafficking, recycling and degradation of EGFR have been extensively investigated, very little is known as to whether EGFR escapes lysosomal degradation and is instead selectively released extracellularly via exosomes. *In vitro* studies have now shown that exosomes isolated from immune and cancer cells contain EGFR, EGFR ligands and soluble isoforms of EGFR. Additionally, tumor cells release exosomes and/or exosomal cargo into the blood circulation of cancer patients [24–29]. These observations have led us to hypothesize that EGFR could be selectively released via exosomes and may very well play a role in PCa progression. Furthermore, the possibility that the selective uptake of EFGR into exosomes may be, at least in-part, responsible for failure of clinical outcome cannot be overruled, however no comparative analysis between the exosomal contents and tumor cell has been done in this manuscript. To determine whether PCa derived exosomes contain EGFR, we isolated and characterized exosomes from a panel of PCa cells as well as serum from LNCaP xenografted mice and serum/plasma from PCa patients. This is a first report showing that EGFR is contained in the exosomes derived from PCa cell lines, both LNCaP xenograft and PCa patient serum. These observations are encouraging to further investigate the possible role of EGFR-containing exosomes in pro-survival and treatment resistance mechanisms as well as potential biomarkers in PCa diagnosis and progression.

## Materials and Methods

### Ethics Statement

Frozen PCa patient plasma/serum was purchased from a private blood and tissue repository, Bioserve’s Global Biorepository, 9000 Virginia Manor Road, Suite 207 Beltsville, MD 20705 USA (http://www.bioserve.com/human-samples/global-biorepository-overview.cfm). The control serum was obtained from 31 year old healthy male volunteer with a verbal consent approved by the ethics board (certificate #H09-01010). The University of British Columbia Clinical Research Ethics Board (certificate #H09-01010) approved the use of commercially acquired human serum to be used for the purpose of this research.

The approval for animal work was obtained from the University of British Columbia’s Institutional Animal Care Committee (IACC, # A11-0337). During the study the care, housing and use of animals was performed in accordance with the Canadian Council on Animal Care Guidelines and all efforts were made to minimize the suffering.

### Cell Culture

Human prostate cancer cells, LNCaP [30]and C4-2 cells were maintained in RPMI 1640 medium whereas DU145 and PC3 in Dulbecco’s Modified Eagle’s Medium (DMEM) supplemented with 5% FBS (Invitrogen) and antibiotic, at 37°C in 5% CO2. Benign RWPE-1 cells also were grown in keratinocyte-SFM (KSFM) with growth supplement (GIBCO) and 1% penicillin-streptomycin (Invitrogen). Cells were grown to 60–70% confluence and serum-starved for 48–72 hours prior to exosomes isolation. All the cell lines were obtained from ATCC.

### Serum samples

Mice bearing LNCaP xenografts were prepared as previously described [31, 32]. Briefly, athymic mice were inoculated with 2 x 10^6^ LNCaP cells. Tumors were grown for 28 days after which serum was derived from blood drawn by cardiac puncture upon euthanisation. Serum samples were collected by from three nude control mice and three mice bearing various sizes of tumors: small (<400mm^3^); medium (400-1000mm^3^) and large (>1000mm^3^). Frozen PCa patient plasma/serum was purchased from a private blood and tissue repository, Bioserve (Beltsville, MD, USA). Four human plasma and three serum samples obtained from PCa patients (Table 1) were analysed in this study. The control serum was obtained from two 31 year old healthy male subjects. Approval was obtained from The University of British Columbia Clinical Research Ethics Board for human serum to be used for the purpose of this research.

**Table 1:** Information of PCa plasma and serum used to derive exosomes.

Subject	Medications	Age	
1	None	31	Healthy male
2	Simvastatin 20mg, Glipizide 500mg, Bystolic 10mg, Exforge 5/160	83	Stable Disease State
3	Thyroid Medication; Cholesterol Medication; Lupron	54	Stage 4; Stable
4	Lupron; Mitoxantrone; Mitrofurantoin; Prednisone; Synthroid (on and off)	75	Flare-Up: Progressive disease in pelvis
5	Lupron; Mitoxantrone; Mitrofurantoin; Prednisone; Synthroid	75	Flare-up
6	ASA, Multivitamins, Lisinopril, Metropolol, Zocor	69	T1C:Remission
7	Metoprolol; Lovastatin; Calcium; Vitamin D; Aspirin; Omeprazole	83	Chemically Castrated
8	Diovan; Vitamin D; Omega 3; Multivitamins; Aspirin; Simvastatin	81	Unknown

### Antibodies

The antibodies used were: mouse anti- CD-9 (C-4, #sc-13118), mouse anti-Alix (1A12, #sc-53540) and goat anti-EGFR (N-20, #sc-31155) from Santa Cruz Biotechnology Inc, (Santa Cruz, CA); rabbit anti-LAMP2 (#ab37024) from Abcam (Toronto, ON) and rabbit anti-GRP94 (#2104) from Cell Signaling Technology (Whitby, ON). All primary antibodies were used at a concentration of 1:1000 for western blot analyses. The secondary antibodies used for detection were Alexa Flour 680 donkey anti-rabbit (# A10043, 1:10000), donkey anti-mouse (#A10038, 1:10000) and donkey anti-goat (#A21084, 1:10000) from Life Technologies (Invitrogen), Burlington ON.

### Preparation of exosomes fractions

Exosomes from PCa cells were isolated using serial centrifugation method as previously described [9]. Frozen patient plasma/serum or LNCaP xenograft serum samples were diluted with phosphate buffer saline (PBS) in a 1:1 ratio [33]. Plasma/serum samples were then pre-treated with anti-IgG antibody (1:500 dilution) coupled to A/G sepharose beads (25μl) to precipitate excessive non-specific immunoglobulins. After an overnight incubation at 4°C, the pre-treated samples were centrifuged at 5000x*g* for 15 min and the pre-cleared serum was used for exosomes isolation using a standard ultracentrifugation method previously reported [9, 33]. Briefly, the pre-cleared serum samples went through a series of centrifugation steps (2000x*g* for 20 min and 20,000x*g* for 45 min) and then transferred onto 30% sucrose solution, followed by ultra-centrifugation at 110,000x*g* for 2 h. Isolated exosomes were recovered from the sucrose solution and stored at -80°C until further analyses.

### Transmission electron microscopy (TEM)

Exosomes isolated from LNCaP xenograft serum and PCa patient plasma/serum were adsorbed onto glow discharged 300 mesh formvar/carbon-coated TEM grids (Ted Pella, Redding California, USA) for 5 min. The samples were negatively stained with 2% aqueous uranyl acetate for 5 min and observed with a Hitachi H7600 TEM operated at 80kV (Hitachi High-Technologies Corp., Tokyo, Japan). Images were captured with a side mounted 1K AMT Advantage digital camera (Advanced Microscopy Techniques, Corp. Woburn, MA, USA) [9].

### NanoSight^™^ particle tracking analysis

The size and concentration of the isolated exosomes were analysed using the NanoSight^™^ LM10-HS10 system (NanoSight Amesbury, UK) as recently described [34]. Each exosomes sample was diluted in exosomes-free pre-filtered PBS to obtain measurable concentration between 0.5×10^8^ and 5×10^9^ particles/ml. For analysis, a monochromatic laser beam (405nm) was applied to the diluted plasma exosomes that was injected into a LM12 viewing unit using a controlled syringe system laser. NanoSight^™^ tracking analysis (NTA) software version 2.3 analyzed the samples at a constant temperature (25°C) with camera shutter speed at 60 milliseconds and gain set to 1400. The NTA software produced six videos of 60 seconds duration, with a 5-second delay between the recordings creating six replicate histograms that were averaged to give the final estimate of the particle size and concentration of exosomes. NTA settings were pre-optimized and kept constant between samples.

### Western blot analysis

Exosome samples were processed in radioimmunoprecipitation assay (RIPA) buffer to release the exosomal content. Protein concentration was then measured by using the BCA Protein Assay kit according to manufacturer’s instructions (Thermo Scientific Pierce). Equal amount of protein (30μg) was loaded for each sample on a 10% SDS-PAGE gel. Exosomal markers were identified using primary antibodies specific for lysosomal-associated membrane protein-2 (LAMP-2) (1:1000), CD-9 (1:1000) and Alix (1:1000). The expression of EGFR in exosomes derived from PCa cell lines as well xenograft or human plasma/serum was detected using goat anti-EGFR antibody which detects EGFR of human and mouse origin.

### ELISA for EGFR detection

EGFR levels in exosomes isolated from LNCaP xenograft and patient plasma/serum were determined using the EGFR ELISA kit (Sigma Aldrich, # RAB0160) according to the manufacturer’s protocol. Lyophilized human EGFR protein standard, whole plasma/serum and exosomes samples were added to the 96-well plate which was pre-coated with anti-human EGFR antibody for 2.5 h at RT. RWPE-1 cell lysate was used as the positive control. Biotinylated EGFR antibody was added for 1 h at RT following washes. HRP–Streptavidin was then incubated for 45 min. ELISA Colorimetric 3,3′,5,5-tetra-methylbenzidine (TMB) was added for 30 min in the dark and 0.2M sulphuric acid (stop solution) was added immediately to stop the reaction upon which yellow colored product was measured at 450nm using an automated ELISA reader (Rayto, RT-1904C Chemistry Analyzer, Atlanta GA, USA) at 450 nm. The results represent amount of EGFR in unprocessed plasma and purified exosomes as ng/ml. The assay was repeated twice and with each sample in triplicate.

### Statistical Analysis

Statistical analysis was performed using GraphPad Prism 6.0. The difference between exosomal EGFR in PCa patients and control subjects was analysed by applying the student t-test. The data are expressed as mean ±SEM.

## Results

### Three fold enrichment of exosomes in PCa patient serum than healthy controls

**Fig 1:**
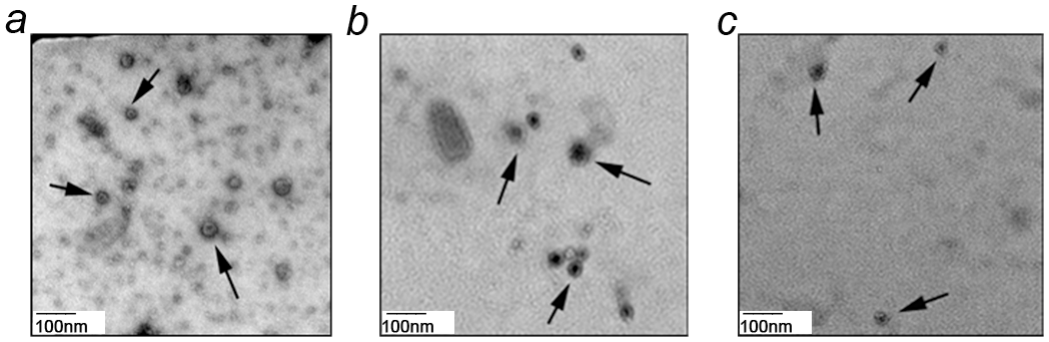
TEM analysis show presence of exosomes. Representative TEM images of exosomes derived from **a)** C42 PCa cell line **b)** LNCaP xenograft serum and **c)** patient plasma by ultracentrifugation method. Exosomes were negatively stained with 2% uracyl acetate after removal of moisture. Arrows indicate cup-shaped structures which are identified as exosomes (30–100 nm in diameter).

**Fig 2:**
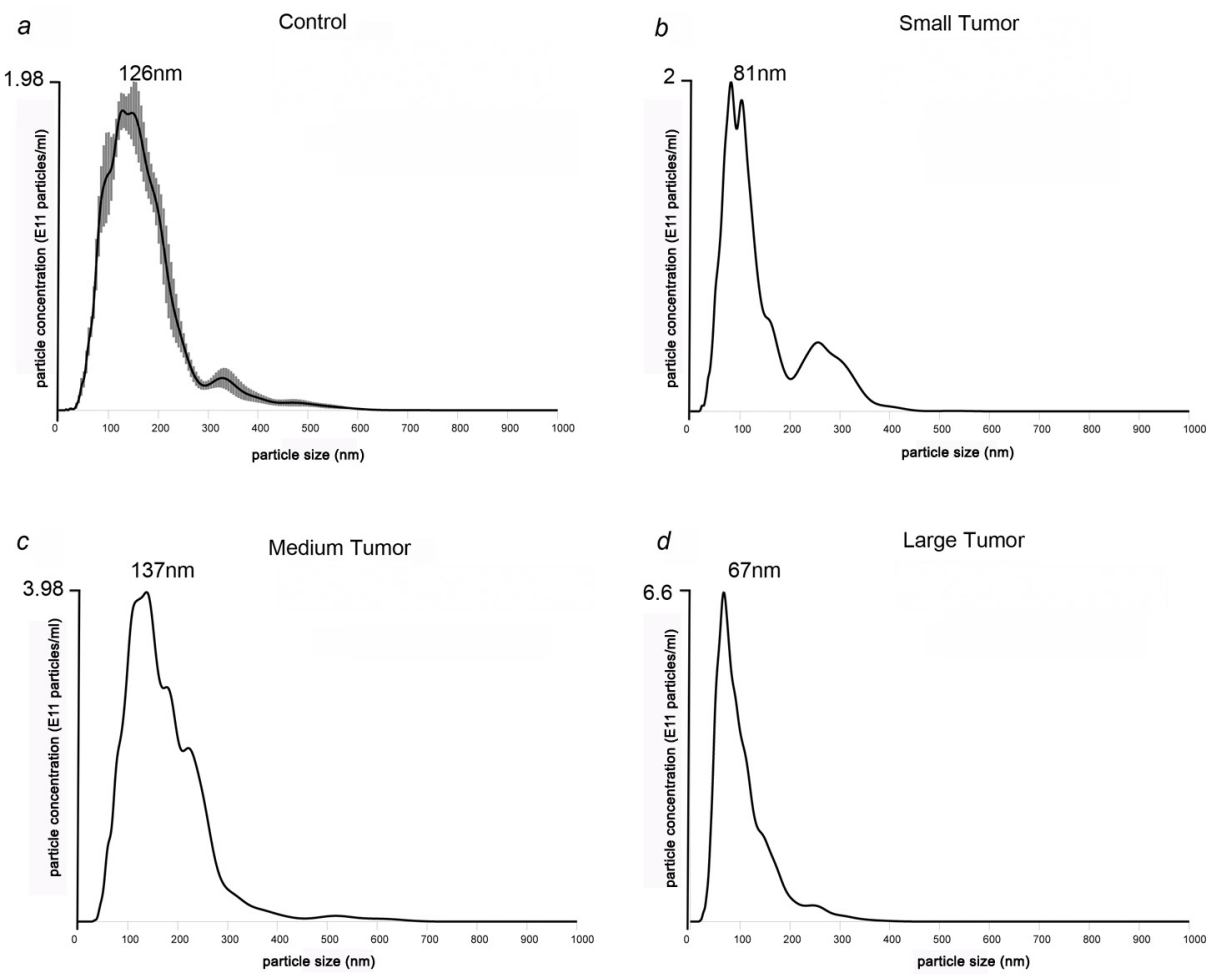
Representative graphs of NanoSight^™^ particle tracking analysis. The analysis showed that mean size of exosomes isolated from control mouse was 126 nm **(a)** whereas LNCaP xenografted mice bearing small tumour was 81nm (b), 137 nm from medium **(c)** and 67 nm from large tumours **(d)**. The concentration of exosomes secreted increased with the increasing size of the tumour.

**Fig 3:**
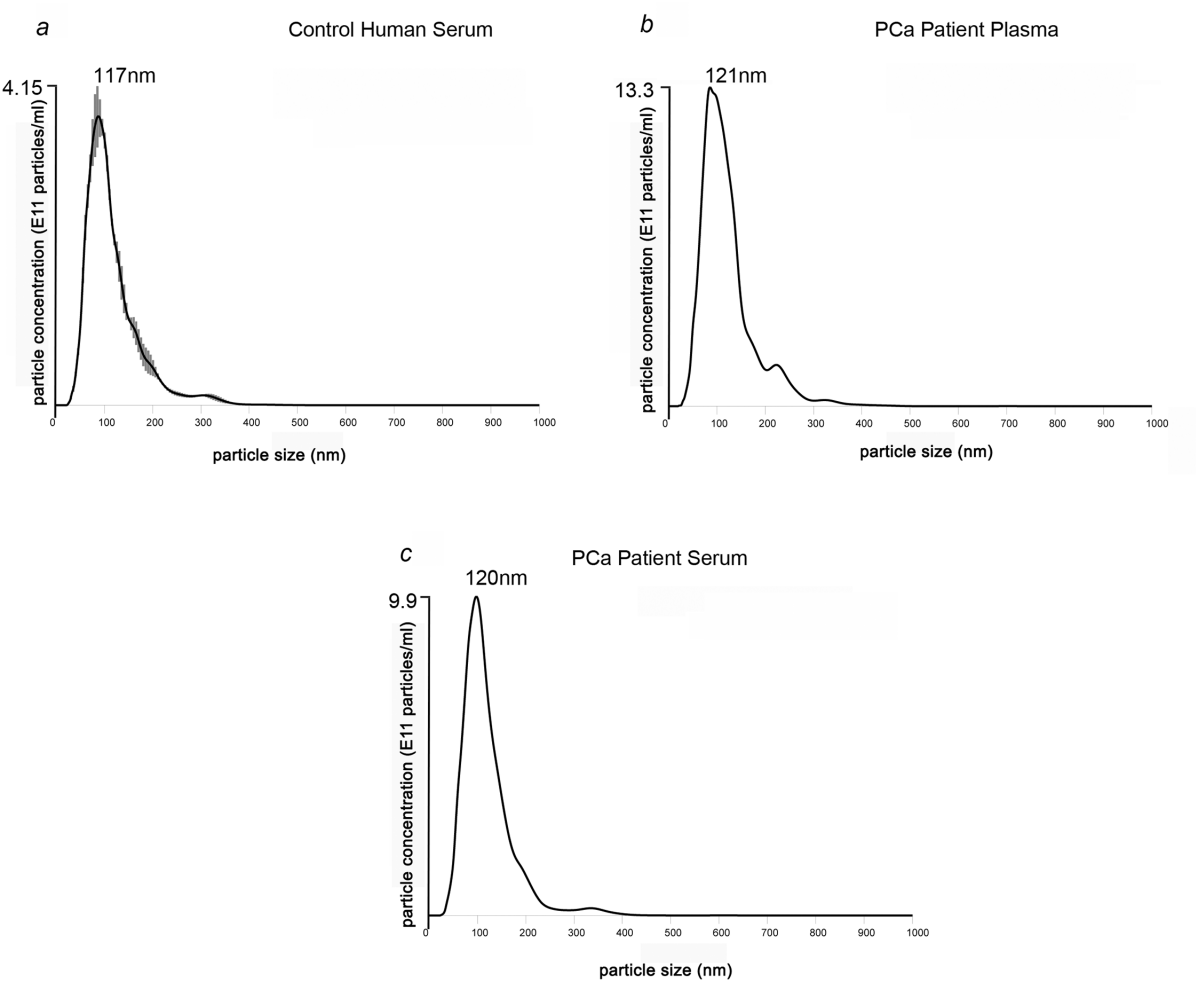
PCa patient serum has higher number of exosomes than control. NTA profiles of exosomes show a single peak that represents mean particle size and concentration of exosomes from normal subject (a) and patient plasma (b) and serum (c). The mean particle size ~120nm nm confirmed presence of exosomes. Exosomes derived from PCa patient was significantly higher than the control human serum.

Characterization of exosomes isolated from LNCaP xenografted mouse serum and PCa patient plasma/serum was carried out using TEM, NanoSight^™^ and Western blot analyses, collectively. TEM analyses revealed a majority of vesicles in the 100nm size range which were characteristic of exosomes, according to their classic cup-shaped morphology, in representative C4-2 PCa cells, LNCaP xenograft serum as well as PCa patient serum (Fig 1). The presence of exosomes was further confirmed using NanoSight^™^ technology as recently described [34]. Figs 2 and 3 showed NTA profiles of exosomes with a single peak that represents the mode size of exosomes (85-150nm), as per the size of exosomes reported routinely in literature. We observed that the total concentration of exosomes derived from control mice serum was 1.98 x 10^11^ particles/ml (Fig 2A). Interestingly the number of particles increased in the serum derived from mice bearing small (2.2 x 10^11^, Fig 2B), medium (3.98 x 10^11^ Fig 2C) and large (6.66 x 10^11^ Fig 2D) tumors suggesting that exosomes are more abundant in tumor bearing mice than the normal mice. Furthermore, we measured the number of exosomes from serum derived from control human and PCa patients. Fig 3A showed that control human serum contained 4.15 x 10^11^ particles/ml, whereas PCa patient plasma (13.3 x 10^11^) and serum (9.9 x 10^11^) showed two-fold increase in number of particles indicating that tumor cells produce more exosomes than the normal cells (Fig 3B and 3C). The NanoSight analyzed between 3000–4000 particles and hence the SEM was low between ±0.12–0.35.

### Identification of markers confirm exosomes isolation from serum

**Fig 4:**
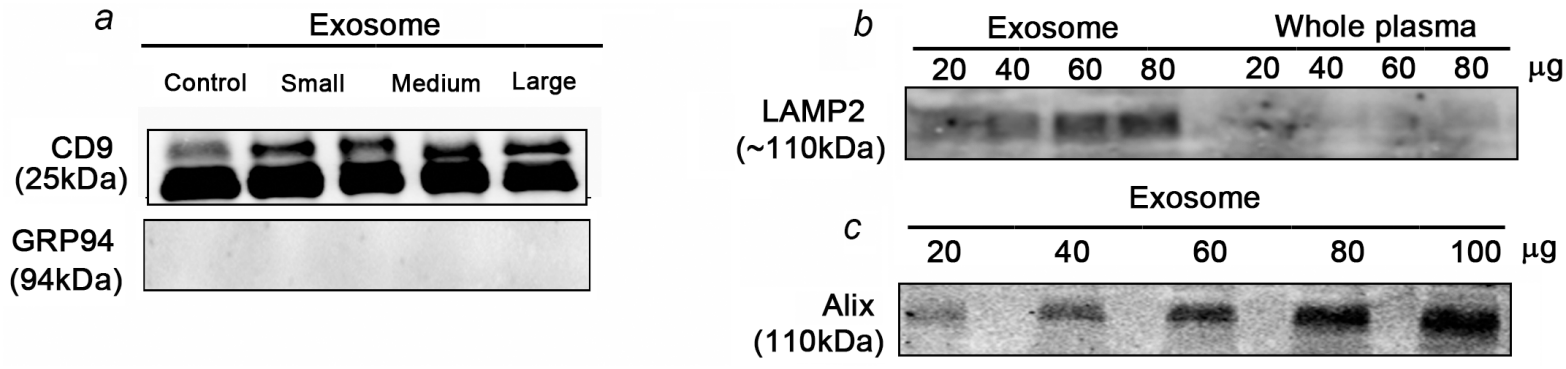
Exosome isolation from plasma is validated by the presence of exosome markers. **a)** CD9 was present in exosomes derived from LNCaP xenograft mice bearing small, medium and large LNCaP tumours whereas the control mouse serum lacked CD9. GRP94, a known endoplasmic reticulum protein which is used as a negative control was absent in the exosomes suggesting enrichment **b)** LAMP2 was present in exosomes derived from PCa patient plasma whereas absence of LAMP2 in whole plasma indicated successful enrichment. **c)** Alix was present in exosomes derived from patient plasma at different exosomal protein concentrations.

Western blot analyses were performed to determine the expression of exosome-specific markers. As shown in Fig 4A, CD-9 (tetraspanin), a commonly used membrane-bound exosomal marker was higher in the serum of small LNCaP xenografts, as compared to that in exosomes from control nude mouse. Interestingly, we did not see a significantly higher expression in the large tumors. These data also correlated with the NanoSight data (Fig 2) showing higher number of exosomes in serum of tumor bearing mice than the control mouse serum. To further validate the purity of the exosome fractions obtained, we determined the presence of a known endoplasmic reticulum marker GRP94 in the exosomes isolated from xenograft serum samples. Our data showed lack of GRP94 in the exosome fractions, indicating successful enrichment and isolation of exosomes from serum samples via sucrose-assisted ultra-centrifugation. The expression of lysosomal-associated membrane protein-2 (LAMP-2) and Alix was also investigated as an alternative exosome marker. Fig 4B and 4C showed that LAMP-2 and Alix was detected in PCa serum/plasma derived exosome fractions and was concentration-dependent. Whereas whole plasma was devoid of LAMP-2, again indicating the quality of exosomes enriched for during isolation.

### PCa derived exosomes contain EGFR

**Fig 5:**
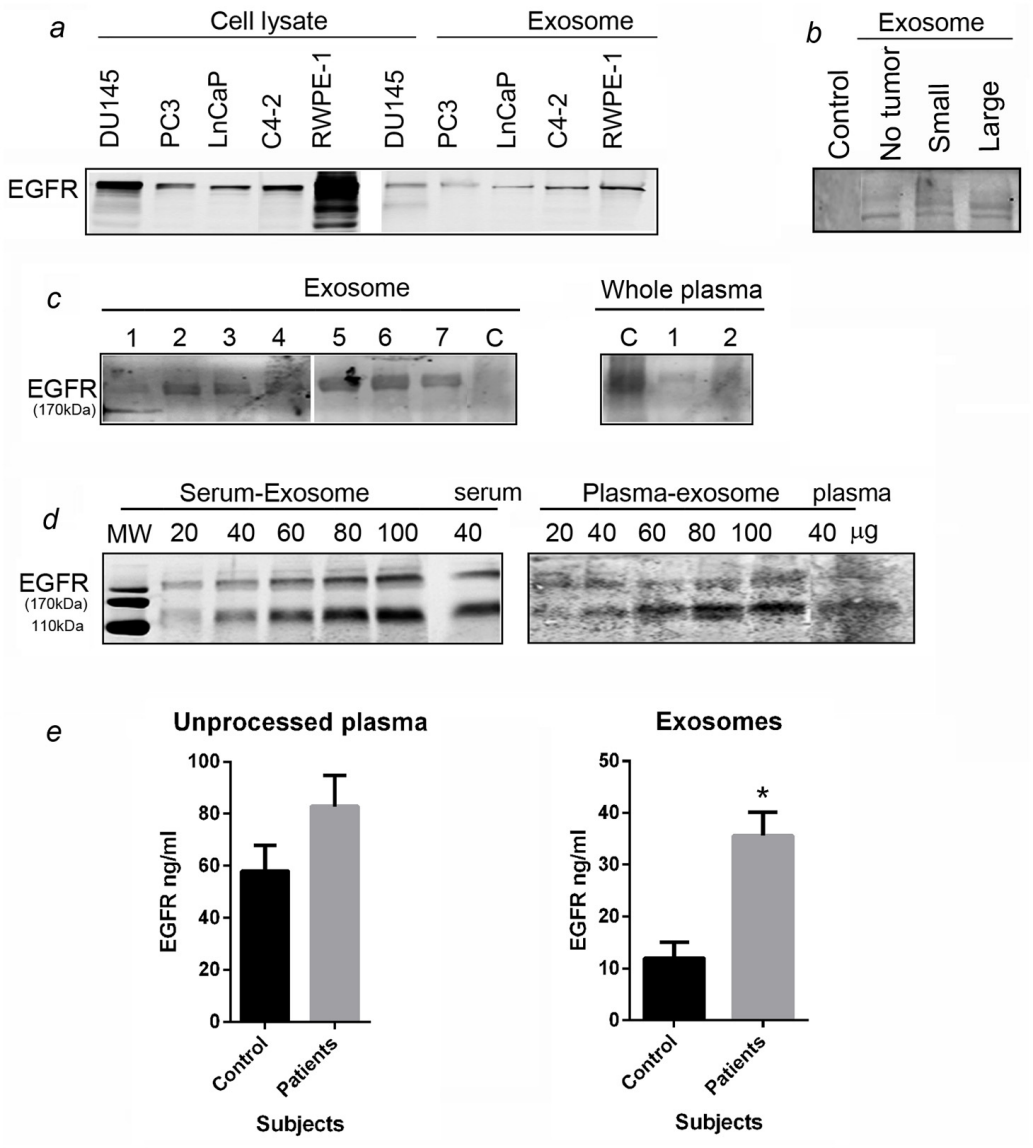
EGFR in PCa derived-exosomes. EGFR was present in exosomes derived from **a) panel** of AR-responsive and AR-unresponsive as well as benign prostate epithelial cells (RWPE-1) and compared with cell lysate, **b)** Control nude mouse and LNCaP xenograft serum. **c)** EGFR is contained in exosomes derived from four different PCa patients’ plasma (1–4), 3 serum samples (5–7) and control subject and in unprocessed plasma from control subject and patient (1 and 2). **d)** The expression of EGFR at 170kDa and 110kDa in serum and plasma is increased with increasing loading protein concentration. Interestingly, there is a significant amount of EGFR in unprocessed plasma which is in addition to the exosomal fraction. The histogram **(e)** shows EGFR levels (ng/ml) measured by ELISA in unprocessed plasma from control subject and PCa patient plasma/serum and exosomes derived from corresponding plasma/serum. The levels of serum EGFR are relatively similar in control and PCa subjects whereas exosomes isolated from PCa patient serum contained significantly higher amounts of EGFR than the control subject. Data represented as mean ±SEM, *p<0.05.

EGFR is a potent cell regulator associated with advanced PCa progression. We next determined whether PCa cells and serum derived exosomes contain EGFR. Fig 5A shows differential expression of full-length 170kDa EGFR in total cell lysates as well as exosomes fractions derived from a panel of AR-positive (LNCaP, C4-2, RWPE-1) and AR-negative (DU145, PC3) cell lines. Overall, the presence of EGFR in total cell lysates is higher than its exosome counterparts, whereas cell-derived exosomes show variable EGFR content for different cell lines. We next determined the presence of EGFR in the *in vivo* samples. Exosomes from control mice serum showed no EGFR however exosomes isolated from serum of LNCaP xenografts contained EGFR irrespective of the presence of tumor and tumor size (Fig 5B).

In order to validate the clinical relevance of exosomal EGFR, we examined whether exosomes isolated from PCa patient plasma/serum contained EGFR. Western blot analyses showed detectable levels of a full-length 170kDa EGFR in two out of four exosome fractions of the PCa patient plasma and all three serum samples, whereas the exosomes from control samples were negative for EGFR (Fig 5C). As shown in Fig 5D, exosomes contained EGFR in a concentration dependent manner. More interestingly, the unprocessed whole serum also showed considerable levels of EGFR suggesting the presence of soluble form EGFR circulating in the serum of PCa patients as was previously reported for pancreatic cancer patient serum in a study exploring exosomes in serum from this patient population[35]. Furthermore, EGFR levels were detected in whole plasma/serum and exosomes derived from PCa patients and control subject using ELISA assay (Fig 5E). ELISA measurement showed that soluble EGFR levels in patient plasma or serum were not remarkably different than the control serum; however, EGFR levels were significantly higher in exosomes derived from PCa patients’ plasma/serum when compared with the control subject.

## Discussion

Exosomes circulating in blood may provide important information that may be often neglected in prognostic evaluations. Our lab recently published an extensive proteomic and lipid analysis of exosomes derived from PCa cells *in vitro* with a view to providing insight into candidate protein biomarkers which exosomes have the potential to yield [9]. We have also established a reproducible method for isolation and quantification of exosomes from human serum[34]. The release of exosomes in blood, urine and other biological fluids thus offers an opportunity to utilize non- invasive methods for prognostic and diagnostic evaluation of PCa in patients. This work, focusing on relevance of EGFR in PCa, describes the isolation and characterization of exosomes derived from *in vitro*, *in vivo* and clinical PCa samples as an extension of our previous work in PCa cell lines[9] [34].

Our study demonstrates, the presence of EGFR in exosomes purified from PCa cells cultured *in vitro*, serum of LNCaP xenograft bearing mice and PCa patient serum. Several reports have indicated the presence of EGFR in exosomes derived from glioblastoma, pancreatic, breast cancer and ovarian cell lines *in vitro* and in exosomes from lung cancer patient serum, however limited is known of EGFR in PCa derived exosomes[36–39]. EGFR is often over-expressed and is associated with aberrant signaling leading to aggressive malignancies and poor patient survival rate [40]. In PCa, hyperactivity of EGFR is linked with androgen independence and metastasis of prostate cancer cells [41, 42]. Since exosomes are involved in cell- cell communication that alters the phenotype of the recipient/target cells [43–45], exosomal-EGFR expressed by various tumor types, including PCa may play an important role in cancer progression. We observed that the number of exosomes was significantly higher in PCa serum and xenograft than the control, suggesting that exosomes may present a tool to differentiate the normal and disease state. Our results are in agreement with a recent study by Turay et al where they show that exosome numbers specifically increased in the serum of PCa patients compared to their control counterparts [46]. Tumor-exosomes are thought to have role in the development of resistance to anti-tumor therapies [47, 48]. However while pre-clinical data indicate that EGFR plays a significant role in PCa progression [21]; clinical trials involving EGFR inhibitors (Gefitinib, Lapatinib or Erlotinib) have shown limited benefit to PCa patients [22, 23, 49].

**Fig 6:**
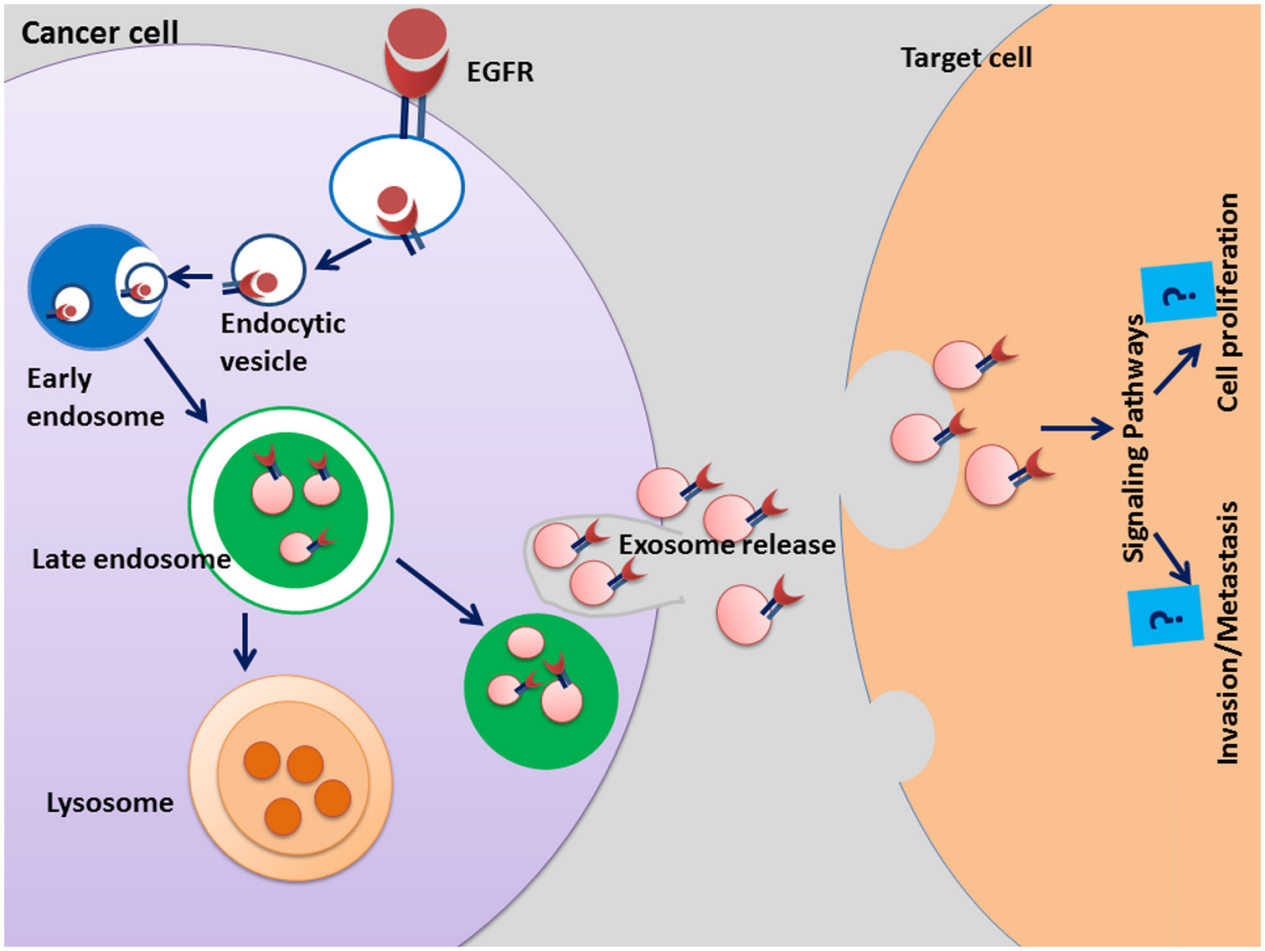
Schematic representation of possible role of EGFR-exosomes in cancer progression. Ligand binding induces rapid activation and internalization of EGFR and endocytosis. Whether EGFR escapes lysosomal degradation and is released extracellularly via exosomes is unknown. The transfer of EGFR via exosomes may significantly alter the tumor microenvironment and could be relevant to progression of an aggressive PCa.

Soluble isoforms of EGFR (sEGFR) have also been identified in conditioned media of breast, non-small cell lung cancer and pancreatic cancer cells as well as circulating in bloodstream [35, 50, 51]. However, there is limited information about the mechanistic role and biochemical properties of these isoforms. There is evidence that full length EFGR undergoes proteolytic cleavage and novel isoforms [52, 53]. Furthermore, a full-length receptor and a 65kDa soluble isoform have been identified on exosomes released from human keratinocyte cell lines [50]. Similarly, we observed a full length 170kDa EGFR and a band at 110kDa in patient plasma and serum-derived exosomes suggesting that soluble isoforms of EGFR may be present in exosomes in addition to the 170kDa EGFR. Interestingly, in PCa cell lines and LNCaP xenograft bearing mouse serum such bands were not observed. Our observation is supported by a similar study in breast cancer cells and patient serum which speculates the presence of soluble growth factors circulating in the patient serum [54]. We also detected a band at 110kDa in patient plasma/serum that could possibly be the free soluble isoform of EGFR. Previous reports showing soluble EGFR protein in serum of patients with metastatic breast and lung cancer could support our results [39, 54]. Although in breast cancer patients, the soluble EGFR isoforms were associated with short survival rate, some imply increased sEGFR or no difference between healthy individuals and patients [39]. Nevertheless, estimation of sEGFR levels in exosomes and peripheral blood may provide clinical relevance in metastatic cancers. The EGFR isoforms which are present in PCa patient serum clearly have an unidentified role in tumorigenesis and further quantitative assays are needed for evaluation of these isoforms in PCa-derived exosomes. In summary, the present study shows exosomes are abundantly secreted in the serum of xenografted mice bearing tumors and PCa patients than their control counterparts. Furthermore, this study identified EGFR in secreted exosomes and provides compelling evidence that circulating EGFR may be constitutive in exosomes and further evaluation of exosomes EGFR in PCa patient serum may identify its role in the resistance of EGFR targeted therapies tested in the clinic against PCa progression (Schematic representation of role of EGFR as shown in Fig 6).

## Supporting Information

S1 Checklist NC3Rs ARRIVE Guidelines Checklist. (DOCX)

S1 Fig Western blot analysis showing CD9 expression in exosomes derived from LNCaP xenograft mice bearing small, medium and large LNCaP tumours whereas the control mouse serum lacked CD9. (TIF)

S2 Fig Western blot showing full uncropped gel of GRP94. The exosomes lacked the presence of GRP94 confirming the isolation successful.
(JPG)
