# Correction: Epidermal Growth Factor Receptor in Prostate Cancer Derived Exosomes

**DOI:** 10.1371/journal.pone.0157392

**Published:** 2016-06-13

**Authors:** Geetanjali Kharmate, Elham Hosseini-Beheshti, Josselin Caradec, Mei Yieng Chin, Emma S. Tomlinson Guns

Fig 4A shows expression of CD-9 in small, medium, and large tumor bearing mice. There are erroneously 5 lanes in the figure; the fifth lane was a duplication of large tumor sample from another mouse. Please view the correct Fig 4, without the extraneous lane in panel A, here.

**Fig 4:**
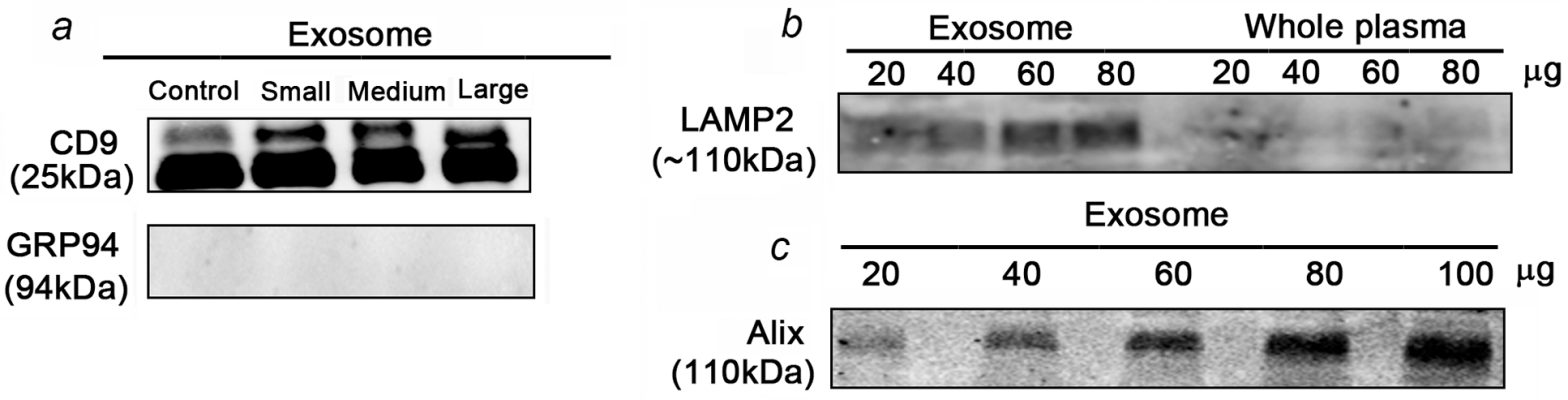
Exosome isolation from plasma is validated by the presence of exosome markers. **a)** CD9 was present in exosomes derived from LNCaP xenograft mice bearing small, medium and large LNCaP tumours whereas the control mouse serum lacked CD9. GRP94, a known endoplasmic reticulum protein which is used as a negative control was absent in the exosomes suggesting enrichment **b)** LAMP2 was present in exosomes derived from PCa patient plasma whereas absence of LAMP2 in whole plasma indicated successful enrichment. **c)** Alix was present in exosomes derived from patient plasma at different exosomal protein concentrations.

## Supporting Information

S1 File Uncropped blot for Fig 4A. (TIF)
