# Supplementary figures and images for: Epidermal Growth Factor Receptor in Prostate Cancer Derived Exosomes
Source: PLoS One. 2016 May 6;11(5):e0154967. doi: 10.1371/journal.pone.0154967 (PMC4859494; doi:10.1371/journal.pone.0154967)

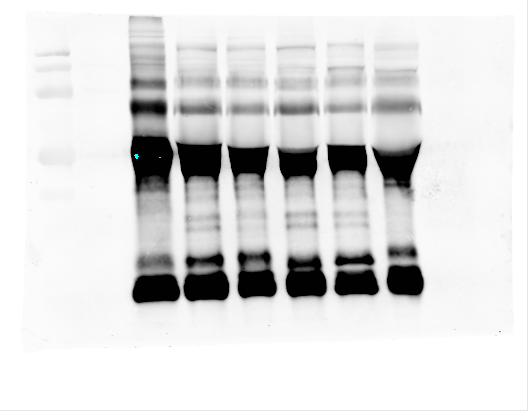

Supplement: S1 Fig — (TIF) [file pone.0154967.s002.tif]

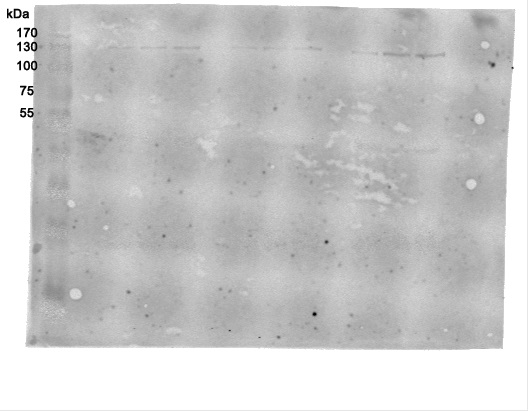

Supplement: S2 Fig — The exosomes lacked the presence of GRP94 confirming the isolation successful. (JPG) [file pone.0154967.s003.jpg]
