# Supplementary figures and images for: Correction: Epidermal Growth Factor Receptor in Prostate Cancer Derived Exosomes
Source: PLoS One. 2016 Jun 13;11(6):e0157392. doi: 10.1371/journal.pone.0157392 (PMC4905623; doi:10.1371/journal.pone.0157392)

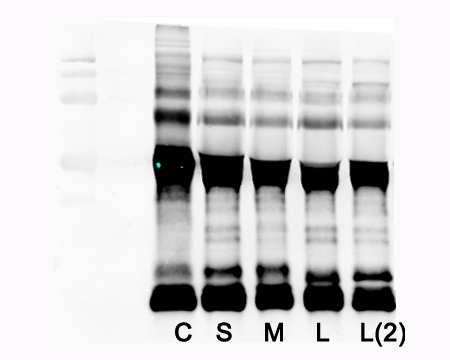

Supplement: S1 File — (TIF) [file pone.0157392.s001.tif]
